# Study on Deposition of Coaxial Electrospinning Fibers by Coaxial Auxiliary Flow Field

**DOI:** 10.3390/polym17030396

**Published:** 2025-02-01

**Authors:** Rongguang Zhang, Xun Chen, Han Wang, Jianfeng Sun, Shize Huang, Xuanzhi Zhang, Jiecai Long

**Affiliations:** 1 State Key Laboratory of Precision Electronic Manufacturing Technology and Equipment, Guangdong University of Technology, Guangzhou 510006, China; 2 School of Electromechnical Engineering, Guangdong University of Technology, Guangzhou 510006, China

**Keywords:** gas-assisted coaxial electrospinning (GACES), coaxial auxiliary flow field, uniformity

## Abstract

Gas-assisted coaxial electrospinning (GACES) is a simple and general method for the mass preparation of coaxial nanofiber membranes, which has great industrial potential. However, in the manufacturing process, due to the bending instability of the jet in the electric field and the pulling effect of the gas flow field, the deposition uniformity of the fiber is still a big problem. Through finite element simulation analysis of the flow field in the manufacturing process and the construction of the jet mechanics model after adding the flow field, the influence mechanism of coaxial auxiliary flow on the fiber deposition area and its uniformity was successfully revealed in this research. Finally, the deposition area and thickness uniformity of coaxial fibers are increased by 3 times (the deposition area: 19.63 cm^2^ → 78.50 cm^2^) and 2.34 times (the standard variance: 3 μm^2^ → 10 μm^2^) by gas-assisted coaxial electrospinning. At the same time, the coaxial auxiliary gas flow also reduces the coaxial fiber diameter by 36.9% (the average fiber diameter: 241 nm ± 5 nm → 152 nm ± 23 nm) and the distribution range by 66% (the standard variance: 1.5 × 10^2^ nm^2^ → 51 nm^2^). This research provides a reliable idea and experimental basis for homogeneous preparation of coaxial nanofiber membranes.

## 1. Introduction

Coaxial nanofibers have attracted much attention from all fields due to their unique micro-/nanostructure [1,2,3,4,5]. Common coaxial nanofiber fabrication processes include template fibers [6], self-assembly [7], template synthesis [8], coaxial electrospinning [9,10,11,12,13], etc. Among them, coaxial electrospinning is a simple and versatile method for preparing coaxial nanofiber membranes, which can be divided into needleless coaxial electrospinning and needle coaxial electrospinning depending on the spinneret [14,15,16,17]. However, so far, the electrospinning technology that can prepare coaxial nanofibers on a large scale is still subject to many limitations. Gas-assisted electrospinning is a new technology for the preparation of nanofibers that has emerged in recent years and has the advantage of efficient large-scale preparation of nanofibers [18,19]. Therefore, the gas-assisted coaxial electrospinning (GACES) technology has great industrial application potential in the large-scale preparation of coaxial nanofibers, but it has received less attention and has been developed slowly.

Meanwhile, current methods for controlling coaxial nanofiber deposition mainly rely on simple and inaccurate techniques that vary electrospinning time, which often produce coaxial nanofiber membranes of non-uniform thickness. Moreover, the bending instability of the jet may also lead to the non-uniform deposition of nanofibers, resulting in local thin regions of the coaxial nanofiber membrane [20,21]. This non-uniformity can reduce the functionality of coaxial nanofiber membrane as a filter or scaffold, as it can affect the filtration efficiency or mechanical properties, affecting the application of electrospinning coaxial membranes in various fields [22,23]. So, in order to achieve uniform deposition of coaxial fibers in electrospinning, it is necessary to research the fiber deposition in the gas-assisted coaxial electrospinning process.

The objective of this research is to investigate the effect of coaxial gas flow on the deposition uniformity of coaxial nanofibers. Through the finite element simulation analysis of the gas-assisted coaxial electrospinning process and the construction of the mechanical model of the gas-assisted coaxial electrospinning jet, the influence mechanism of the coaxial flow field on the deposition of coaxial fibers was successfully revealed. At the same time, the quantitative analysis of the experimental results was carried out to realize the quantitative characterization of the fiber deposition area, deposition thickness uniformity, fiber average diameter, and distribution interval under different gas field parameters. Finally, the deposition area and thickness uniformity of coaxial fibers are increased by 3 times and 2.34 times by gas-assisted coaxial electrospinning. At the same time, the coaxial auxiliary gas flow also reduces the coaxial fiber diameter by 36.9% and the distribution range by 66%.

## 2. Materials and Methods

### 2.1. Materials

Polyvinyl alcohol (PVA-220, Kuraray, Tokyo, Japan) and polyethylene oxide (PEO-N3000) were obtained from Aladdin Chemical Co. (Shanghai, China). All reagents were of analytical grade and used as received.

### 2.2. Fabrication of Nanofiber Membrane

**Figure 1:**
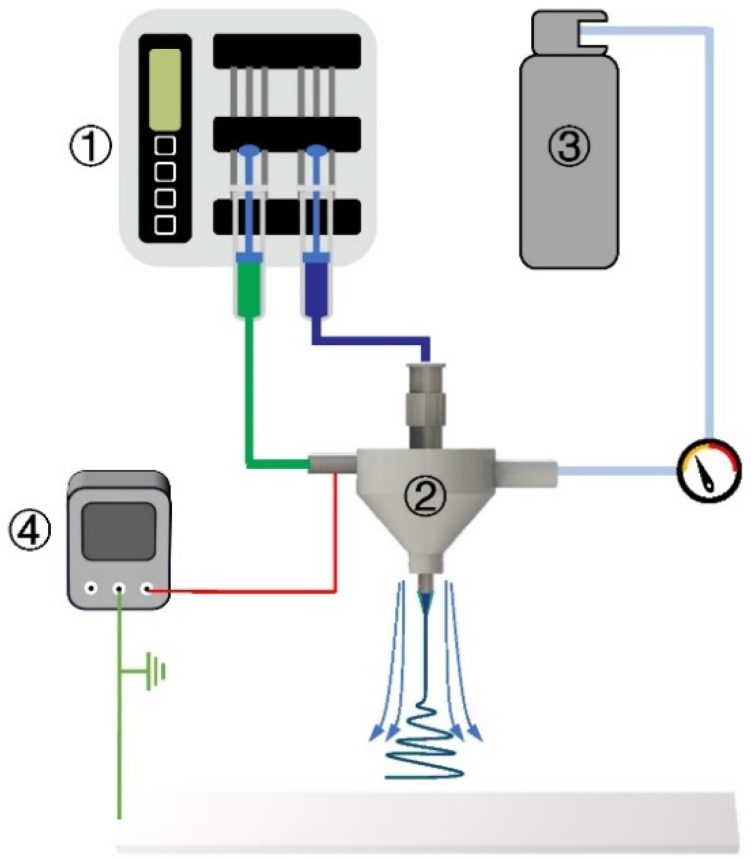
Schematic diagram of coaxial electrospinning by coaxial auxiliary flow field: **①** double-channel pump, **②** auxiliary flow coaxial electrospinning nozzle, **③** compressed gas, and **④** high-voltage power supply.

A 2 wt% solution of PVA and a 6.5 wt% solution of PEO in ion water were prepared. The experimental device is self-built, as shown in Figure 1. In this research, we selected a coaxial gas duct nozzle with an inner diameter of 2.78 mm and a coaxial electrospinning needle with an inner/outer diameter of 1.6/2.1 mm and 0.7/1.07 mm, respectively. The needle was charged to 15 kV using a power supply, and the PEO shell solution and PVA core solution were pumped from the gas-assisted coaxial electrospinning nozzle at a rate of 1.6 mL/h and 0.1 mL/h, respectively, via a double-channel pump. The pressure regulating valve control of auxiliary gas flow pressure is set for 0/0.01/0.02/0.03/0.04/0.05 MPa, and the needle was positioned 25 cm above a grounded collector. A jet of the polymer solution was launched from the needle tip and was collected on the grounded surface, and an ultra-fine coaxial fiber membrane was collected on the collector.

### 2.3. Characterization and Deposition Evaluation of Nanofibers

The surface morphology of the nanofibers was observed by scanning electron microscopy (SEM, TESCAN MIRA3, Brno-Kohoutovice, Czech Republic). The distribution of nanofiber diameters was calculated from the SEM images using ImageJ software 1.51W (NIH, Bethesda, MD, USA). We randomly selected 100 nanofibers from the SEM images of each nanofiber membrane sample for diameter measurement and analyzed and displayed the diameter distribution of the nanofibers.

### 2.4. Numerical Simulation of Flow Field

COMSOL 6.2 Multiphysics software was used to construct the gas flow field model of an auxiliary flow coaxial electrospinning for finite element simulation analysis, and the two-dimensional lines with different axis distances were parametrically scanned to obtain the relevant gas flow field velocity distribution data set.

## 3. Results and Discussion

### 3.1. Theoretical Research of Electrospinning Instability by Introducing Coaxial Gas Flow

#### 3.1.1. Simulation Analysis of Flow Field in Auxiliary Flow Coaxial Electrospinning

By using COMSOL 6.2 Multiphysics software, the gas flow field model of auxiliary flow coaxial electrospinning was constructed and simulated.

**Figure 2:**
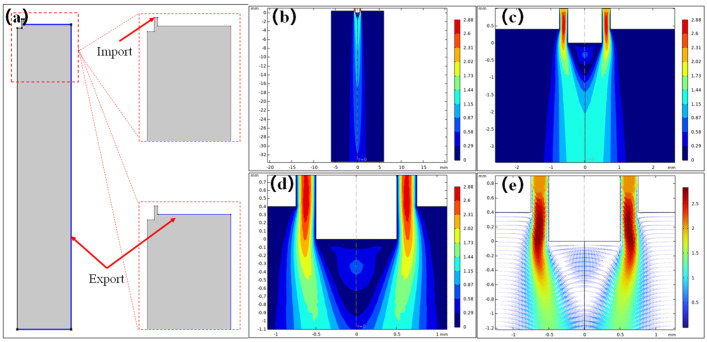
Diagram of flow field model establishment and simulation results: (**a**) boundary condition settings of the gas flow import and export; (**b**) overall velocity distribution cloud map; (**c**,**d**) local velocity distribution cloud map at different scales; (**e**) speed vector diagram of the needle tip.

As shown in Figure 2a, the boundary conditions of the gas flow field are set. The gas flow import is set to the normal flow rate of 2 m/s. The gas flow export condition is set to 0 Pa. The resulting flow speed distribution cloud map and speed vector diagram are presented in Figure 2b–e. From Figure 2b–d, we can see that the overall flow rate gradually decreases along the negative direction of the Z axis from the nozzle, while the action range of the gas flow gradually increases. At the same time, it can be seen from Figure 2e that at the front of the needle, there was an obvious reverse flow phenomenon. This phenomenon had also been reported in the paper of the predecessors [24,25].

**Figure 3:**
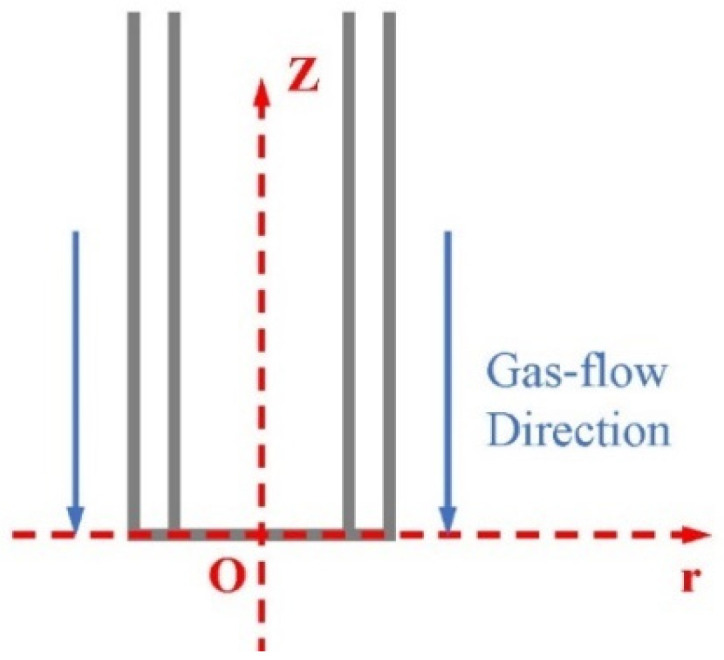
A coordinate system diagram of the gas flow at the needle.

As shown in Figure 3, the gas flow is sprayed from the +z direction to the zero point. To create the coordinate system, the center point of the needle outlet is the center point, and +z and +r are defined as the positive directions of the velocity of the axial and radial flow fields, respectively.

**Figure 4:**
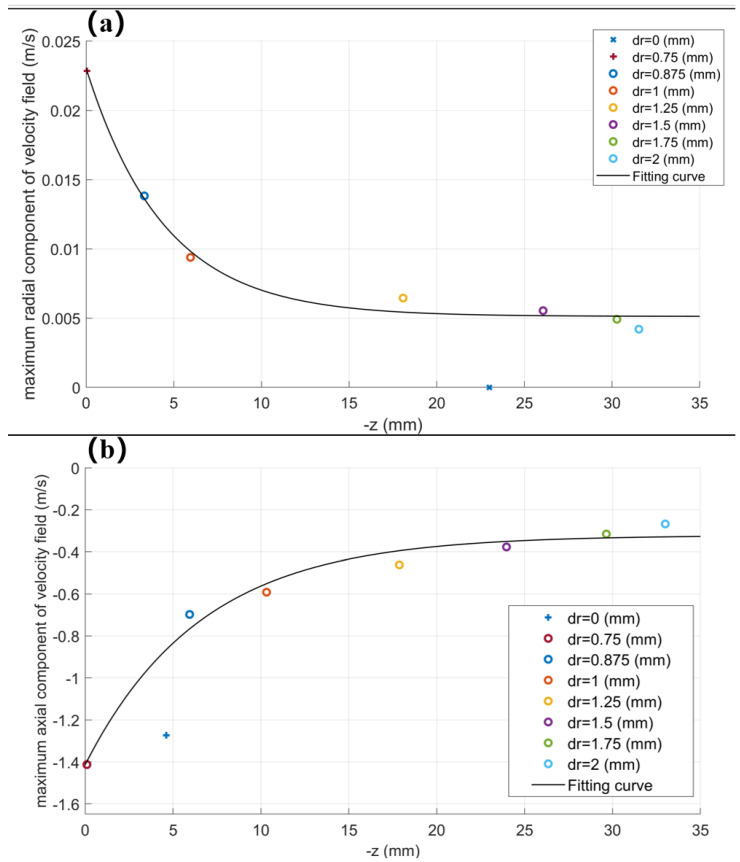
The distribution and fitting curve of the maximum axial/radial component of the velocity field in the axial direction: (**a**) variation diagram of maximum radial component of velocity field; (**b**) variation diagram of maximum axial component of velocity field.

In Figure 3, we define the distance with the axis of Dr, and two-dimensional lines were drawn in Dr = 0/0.125/0.25/0.375/0.5/0.625/0.75/0.875/1/1.25/1.5/1.75/2 mm. By collecting the highest point of axial/radial components of the velocity field on the two-dimensional line, the axial/radial components of the velocity field are analyzed statistically, as shown in Figure 4a,b.

From Figure 4a,b, it can be seen that the overall flow field rate at the diffusion boundary of the flow field gradually decreases with the diffusion. By fitting the data, the following relationship can be obtained:

(1) $$\left\{\begin{array}{c} {V_{z}\propto e}^{-0.2z}V_{o}+100\;\\ V_{r}\propto {0.01e}^{-0.2z}V_{o}+1 \end{array}\right.$$

where $V_{z}$ (m/s) and $V_{r}$ (m/s) represent the axial/radial components of the gas field velocity, respectively, $V_{o}$ (m/s) is the initial velocity of gas flow import, and z (mm) is the axial coordinate.

#### 3.1.2. Mechanical Modeling and Analysis of Auxiliary Flow Coaxial Electrospinning Jet

With reference to the one-dimensional steady model [26,27], the motion process of the electrospinning jet is analyzed. Under the action of an electric field, the charged jet accelerates along a straight line, and the viscous resistance increases. When the viscous resistance is greater than the electric field force, the jet enters the unstable stage, the acceleration of the jet becomes zero, and the small disturbance in the gas will cause the jet to deviate from the equilibrium position. Therefore, the motion radius of the jet is an important parameter to characterize the degree of instability.

**Figure 5:**
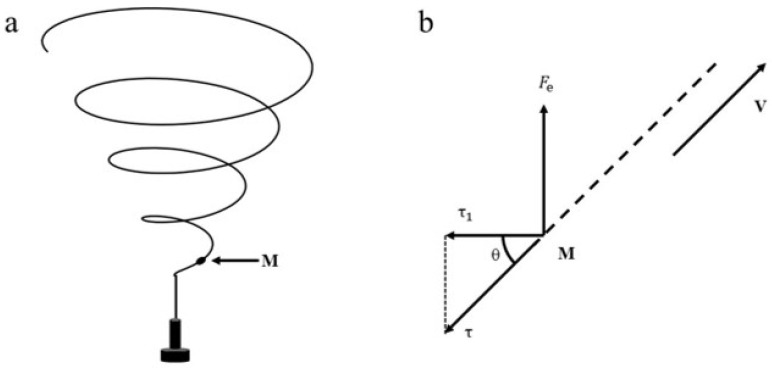
Force analysis of spinning jet without gas field: (**a**) electrospinning jet without gas field diagram; (**b**) force analysis diagram.

In Figure 5a, the motion behavior of any particle M in the unsteady phase of the jet can be divided into linear motion in the vertical direction and approximate circular motion in the horizontal direction. Force analysis is carried out on particle M, as shown in Figure 5b. Based on the centripetal force equation, Newton’s second law of motion, and the relevant force decomposition rules, we can obtain Equation (2) as follows:

(2) $$\left\{\begin{array}{c} \begin{array}{c} F_{n}=\frac{mV^{2}}{R}\\ \tau =AV+BV^{2}\\ \tau_{1}=\tau \sin\theta \end{array}\\ \tau_{1}=F_{n} \end{array}\right.$$

where $F_{n}$ represents the centripetal force of jet motion, m represents the mass of the jet particle, V represents the velocity of particle M, R represents the motion radius of the jet, τ represents viscous resistance, A and B are constants, $\tau_{1}$ is the component force of τ in the radial direction, and θ is the angle between τ and $\tau_{1}$.

From Yu-Qin Wan et al.’s conclusion [28], we can obtain Equation (3):

(3) $$\left\{\begin{array}{c} V\propto r^{-2}\\ r\propto z^{-\frac{1}{4}} \end{array}\right.$$

where r is the motion radius of the jet, which is also R (mm) in this research, and z is the axial coordinate.

Equation (4) can be obtained by combining Equations (2) and (3).

(4) $$R\propto \frac{1}{z^{-\frac{1}{2}}+1}$$

**Figure 6:**
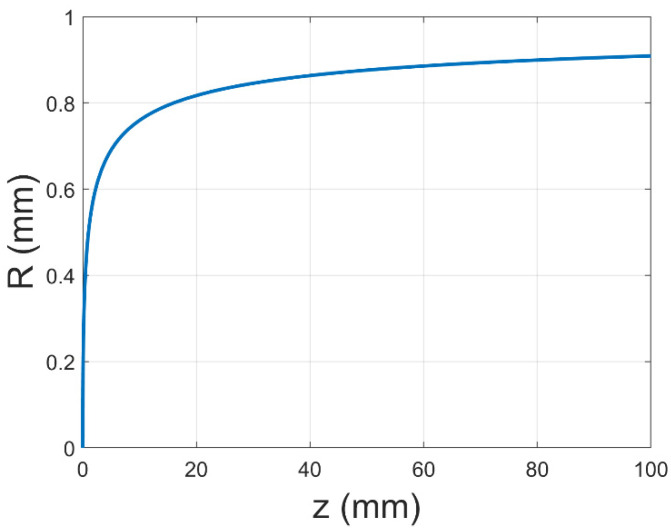
The trend diagram of the relationship between the jet radius (R) and the axial coordinate (z) without a gas field.

Therefore, Equation (5) gives the relationship between the radius of the motion of the jet (R) and the axial coordinate (z) without a gas field, and the trend diagram is shown in Figure 6. This relationship demonstrates that the fiber after the draft of the jet has a fixed deposition region and the size of the region is related to the axial coordinate. This conclusion is consistent with the experimental process captured by Reneker et al. through high-speed cameras [29].

**Figure 7:**
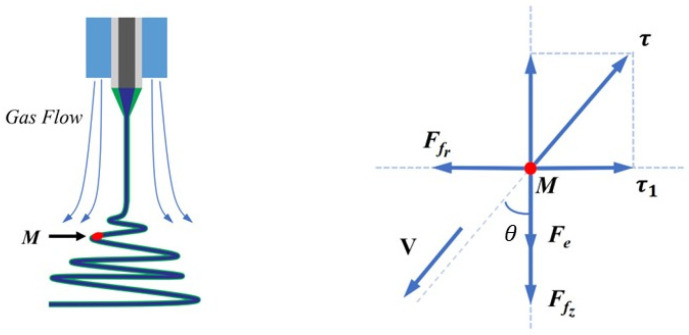
Force analysis of spinning jet with gas field.

When the gas field force is involved, particle M is affected not only by viscous resistance and electric field force, but also by gas field force. Here, the magnitude and direction of viscous resistance and electric field force remain unchanged, as shown in Figure 7.

Similarly to Equation (2), we can obtain Equation (5) as follows:

(5) $$\left\{\begin{array}{c} \begin{array}{c} F_{n}=\frac{mV^{2}}{R_{0}}\\ \tau =AV+BV^{2}\\ \tau_{1}=\tau \sin\theta \\ F_{f_{r}}=\frac{1}{2}\rho S{V_{r}}^{2} \end{array}\\ \tau_{1}-F_{f_{r}}=F_{n} \end{array}\right.$$

where $R_{0}$ (mm) represents the ideal motion radius of the jet, $F_{f_{r}}$ represents the radial component of the gas field force, ρ and S are constants, and $V_{r}$ represents the radial component of the gas field velocity.

Equation (6) can be obtained by combining Equations (4) and (5).

(6) $$R_{0}\propto \frac{1}{z^{-\frac{1}{2}}+1-\frac{1}{2}{V_{r}}^{2}z^{-1}}$$

Therefore, from Equation (6), we can know the relationship between the ideal radius of the motion of the jet and the axial coordinate and the radial component of the gas field velocity after the introduction of the gas field. The ideal radius of the motion of the jet continues to increase either when increasing the axial coordinate or radial component of the gas field velocity.

**Figure 8:**
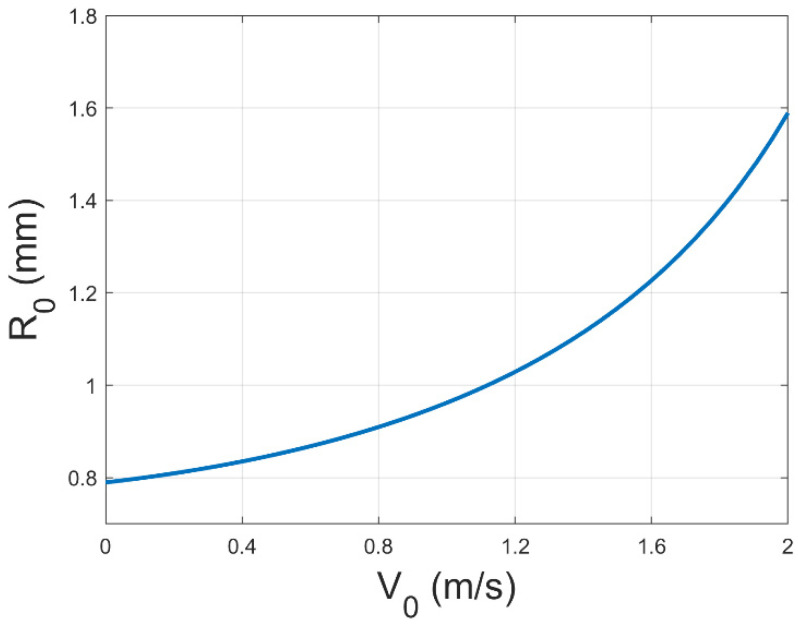
The trend diagram of the relationship between the jet radius R0 and the initial velocity of gas flow $V_{0}$ considering the radial component of the gas field.

Meanwhile, combining these results with Equation (1), we can obtain the trend in *R*_0_ with $V_{0}$ as shown in Figure 8. However, $R_{0}$ is the deposition radius in the ideal situation without the consideration of the spinning height limitation. And this relation equation cannot correctly describe the relationship between the real deposition radius R and $V_{0}$. Therefore, by reintroducing the influence brought by the axial physical field, we can obtain Equations (7) and (9) as follows.

When the axial wind field component is not considered,

(7) $$\left\{\begin{array}{c} \begin{array}{c} z=\frac{1}{2}\bar{a}{\bar{t}}^{2}\\ \tau =AV+BV^{2}\\ \tau_{2}=\tau \cos\theta \end{array}\\ F_{e}-\tau_{2}=m\bar{a} \end{array}\right.$$

where z is the axial coordinate, which is also the spinning distance, $\bar{a}$ represents the axial acceleration without consideration of the axial gas field component force, $\bar{t}$ (s) represents the motion time without consideration of the axial gas field component force, $\tau_{2}$ is the component force of τ in the axial direction, and $F_{e}$ (N) represents the electric field force.

Equation (8) can be obtained by combining Equations (4) and (7).

(8) $$\bar{t}\propto \sqrt{\frac{2}{F_{e}z^{-1}-z^{-\frac{1}{2}}-1}}$$

When the influence of the axial gas field component force is considered,

(9) $$\left\{\begin{array}{c} \begin{array}{c} z=\frac{1}{2}at^{2}\\ \tau =AV+BV^{2}\\ \tau_{2}=\tau \cos\theta \\ F_{f_{z}}=\frac{1}{2}\rho S{V_{z}}^{2} \end{array}\\ F_{e}+F_{f_{z}}-\tau_{2}=ma \end{array}\right.$$

where a represents the axial acceleration when considering the axial gas field component force, t(s) represents the jet motion time after considering the axial gas field component force, $F_{f_{z}}$ represents the axial component of the gas field force, and $V_{z}$ represents the axial component of the gas field velocity.

Equation (10) can be obtained by combining Equations (4) and (9).

(10) $$t\propto \sqrt{\frac{2}{F_{e}z^{-1}+\frac{1}{2}{V_{z}}^{2}z^{-1}-z^{-\frac{1}{2}}-1}}$$

And then, we set a change rate for the jet radius as $\bar{R\;}(mm/s)$, obtained from Equation (11).

(11) $$\bar{R}=\frac{R_{0}}{\bar{t}}$$

Therefore, the radius of the jet after considering the effects of radial/axial physical field can be obtained from Equation (12).

(12) $$R=\bar{R}t$$

Finally, by coupling the above equations, the following can be obtained:

(13) $$R\propto \sqrt{\frac{F_{e}Z^{-1}-{(z}^{-\frac{1}{2}}+1)}{\left[F_{e}z^{-1}+\frac{1}{2}{V_{z}}^{2}z^{-1}-{(z}^{-\frac{1}{2}}+1)\right]{\left[{(z}^{-\frac{1}{2}}+1)-\frac{1}{2}{V_{r}}^{2}z^{-1}\right]}^{2}}}$$

**Figure 9:**
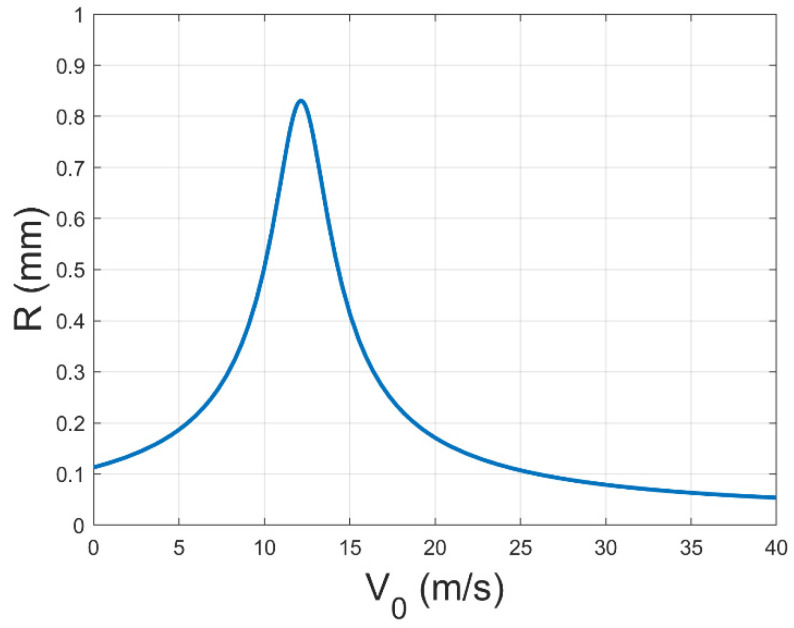
The trend diagram of the relationship between the jet radius R and the initial velocity of gas flow $V_{0}$ considering the radial component of the gas field.

By combining Equation (1), we can obtain a trend in R with $V_{0}$ as shown in Figure 9.

Consequently, the jet, which was originally only affected by the vertical electric field force, was affected by the axial and radial wind forces due to the intervention of the gas field, making the fiber deposition break through the original natural area and the deposition circle larger, which we believe is one of the keys to make the fiber deposition more uniform. However, the axial gas field force causes the jet to accelerate towards the collector, thereby imposing a constraint on the continued increase in the diameter of the deposition circle. Therefore, with an increase in the gas field flow rate, the area of the fiber deposition will demonstrate a tendency to increase and then decrease. So far, the mechanical model of the single nozzle case has been fully described. When the mass production of multiple nozzles is really carried out, the related effects of the flow field and electric field between the nozzles must be considered in the subsequent work.

### 3.2. Experimental Verification

#### 3.2.1. Effect of Coaxial Gas Flow on Coaxial Fiber Deposition Area and Uniformity

**Figure 10:**
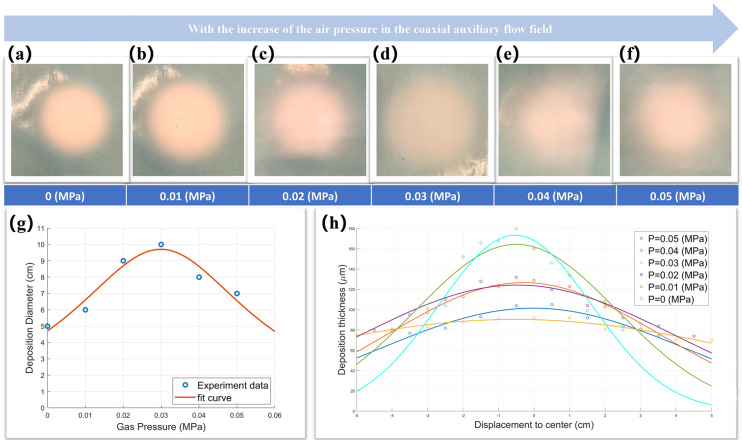
Fiber deposition effect under different gas pressures: (**a**–**f**) fiber deposition circle diagram; (**g**) diameter change and fitting curve of the fiber deposition circle; (**h**) comparison of scatter maps and fitting curves of fiber deposition thickness.

By setting different auxiliary gas pressures, the results of fiber deposition can be achieved, as shown in Figure 10a–f. By measuring the diameter and thickness of the fiber deposition circle, we obtained the scatter diagram of the diameter distribution and thickness distribution of the fiber deposition circle under different pressures, as shown in Figure 10g,h.

In Figure 10a–g, it can be clearly observed that as the gas pressure increases (0 MPa → 0.05 MPa), the diameter of the fiber deposition circle presents a tendency to increase and then decrease (5 cm/0 MPa → 10 cm/0.03 MPa → 7 cm/0.05 MPa). Compared with the results without auxiliary gas flow, the diameter of the fiber deposition circle we are most concerned about increases from 5 cm (0 MPa) to 10 cm (0.03 MPa) at most, which means that the fiber deposition area has increased by three times (19.63 cm^2^ → 78.50 cm^2^). Then, according to Bernoulli’s principle, we can see that, with other parameters constant, the gas pressure is positively correlated with the initial flow rate of the nozzle, i.e., $V_{0}\;\propto \;P$. So, this experimental result verifies the relationship between the real deposition radius R and the initial velocity of gas flow $V_{0}$.

At the same time, according to Figure 10h, it can be seen that when the gas pressure is 0.03 MPa, the diameter of the fiber deposition circle is the largest and the thickness uniformity is the best. In order to further quantify the thickness uniformity, the thickness distribution data of the fiber deposition circle under different gas pressures were simulated by Gaussian fitting, and six fitting curves were compared and analyzed.

The one-dimensional form of the standard Gaussian is shown in Equation (14):

(14) $$y=ae^{-\frac{{\left(x-b\right)}^{2}}{2c^{2}}}$$

where a represents the height of the peak of the curve, b represents the coordinates of the center of the peak, and c represents the standard variance.

As can be seen from Figure 10h, compared to without auxiliary gas flow, the standard variance, which we are most concerned about, increases from 3 μm^2^ (0 MPa) to 10 μm^2^ (0.03 MPa) at the highest, which means that the thickness uniformity increases by 2.34 times.

#### 3.2.2. Effect of Coaxial Gas Flow on Coaxial Fiber Morphology and Diameter Distribution

Due to the addition of coaxial gas flow, the jet fibrosis environment may change. Therefore, we conducted a statistical analysis on fiber morphology and diameter distribution before and after the intervention of coaxial gas flow.

**Figure 11:**
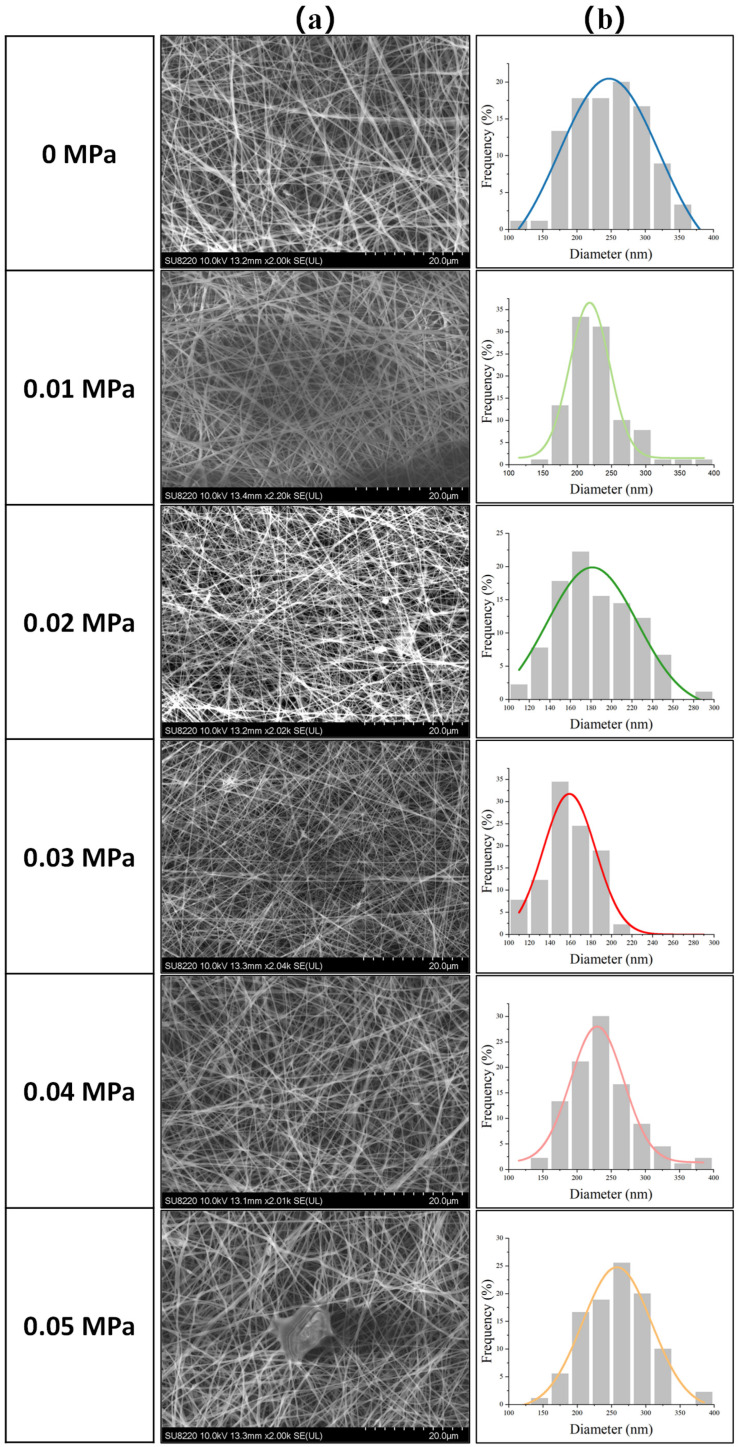
Electrospinning effect under different gas pressures: (**a**) SEM image of fiber morphology; (**b**) nanofiber diameter distribution graph and fitting curve.

**Figure 12:**
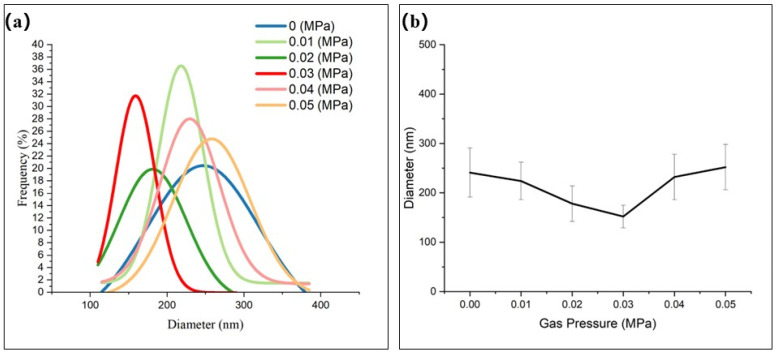
Quantitative analysis of electrospinning effect under different gas pressures: (**a**) the fitting curve of the nanofiber diameter distribution; (**b**) comparison of average diameters.

According to Figure 11a,b, when the gas pressure is lower than 0.05 MPa, the fiber morphology basically maintains a good fiber shape, without the appearance of beads and lumps. And even the fiber diameter becomes thinner, because the addition of gas flow increases the tractive force on the jet, which is conducive to the thinning of the fiber. However, when the gas pressure reached 0.05 MPa, the fiber deposition showed obvious lump and fiber adhesion, which was because the excessive gas pressure made the fiber reach the collector too fast, and the fiber curing was not sufficient. In order to further quantify the change in fiber diameter distribution, Gaussian fitting was performed on fiber diameter distribution data under different gas pressures, and six fitting curves and their average diameters were compared and analyzed, as shown in Figure 12.

(15) $$y=y_{0}+\frac{A}{w\sqrt{\frac{pi}{2}}}e^{-2*{\left(\frac{x-x_{c}}{w}\right)}^{2}}$$

By comparing Equations (14) and (15), it can be seen that a is positively correlated with $\frac{A}{w}$, and b and c are positively correlated with $x_{c}$ and w, respectively. And $y_{0}$ is an auxiliary fitting parameter, so this term will not be compared later.

As can be seen from Figure 12a,b, with the increase in gas pressure, the average fiber diameter decreases first and then increases (241 nm/0 MPa → 152 nm/0.03 MPa → 252 nm/0.05 MPa). Compared to the results without auxiliary gas flow, the average fiber diameter was reduced from 241 nm ± 50 nm (0 MPa) to 152 nm ± 23 nm (0.03 MPa), representing a 36.9% reduction in fiber diameter. The standard variance of the fiber diameter distribution can be reduced from 1.5 × 10^2^ nm^2^ (0 MPa) to 51 nm^2^ (0.03 MPa), which means that the fiber diameter distribution range is reduced by 66%. Unexpectedly, the fiber diameter can be more concentrated in a specific range.

Therefore, by adding suitable coaxial auxiliary gas flow, the fiber deposition area can be increased by 3 times, and the uniformity of fiber deposition thickness can be increased by 2.34 times. At the same time, because the gas flow increases the tractive force on the jet, suitable coaxial auxiliary gas flow can reduce the average diameter of the fiber by 36.9% and the standard variance of the fiber distribution by 65.1%.

## 4. Conclusions

In this research, we simulate the gas flow field in the process of gas-assisted coaxial electrospinning by finite element analysis and establish the jet mechanics model of this process. Through a series of formula derivation and experiments, the influence mechanism of coaxial auxiliary gas flow on the fiber deposition area and its uniformity has been successfully revealed and verified. Finally, in the experiment of gas-assisted coaxial electrospinning, the deposition area and thickness uniformity of coaxial fibers are increased by 3 times (19.63 cm^2^ → 78.50 cm^2^) and 2.34 times (3 μm^2^ → 10 μm^2^), respectively. At the same time, the average diameter and distribution range of coaxial fibers decreased by 36.9% (241 nm ± 50 nm → 152 nm ± 23 nm) and 66% (1.5 × 10^2^ nm^2^ → 51 nm^2^), respectively. Therefore, the addition of a coaxial gas field can effectively increase the deposition area of the coaxial fiber, improve the uniformity of coaxial fiber deposition, refine fiber diameter, and make fiber distribution more concentrated, which provides theoretical guidance and experimental basis for the uniform preparation of coaxial electrospinning fibers. In a follow-up study, we focused on the large-scale preparation of coaxial fibers by gas-assisted coaxial electrospinning. There are still, at least, the following problems that have to be overcome: ① the development of a multi-nozzle module with uniform liquid and gas supply; ② the improvement of gas flow environmental impacts and structural optimization of the multi-nozzle module; and ③ the development of a coaxial nanofiber quality evaluation system and related equipment for large-scale preparation.

## Data Availability

The data presented in this study are available on request from the corresponding author. The data are not publicly available due to [Some of the data relate to the core information of production].
